# The Niagara and Orleans County Veterinary Medical Society

**Published:** 1900-04

**Authors:** Anderson Crowforth

**Affiliations:** Secretary-Treasurer


### THE NIAGARA AND ORLEANS COUNTY VETERINARY MEDICAL SOCIETY.

AN organization meeting was held on April 14, 1900, in the parlors of the Rich Hotel, Middleport, N. Y. Drs. Stocking, Williams, and Crowforth were appointed essayists for the next meeting of the Society, which will be held in Lockport, N. Y., August 11, 1900, and will be known as the annual meeting.

ANDERSON CROWFORTH,
Secretary-Treasurer.

